# Severe Diaphragmatic Eventration Presenting With Recurrent Syncope: A Rare Case Report

**DOI:** 10.1002/ccr3.71473

**Published:** 2025-11-17

**Authors:** Prosper Adjei, Edward Ebo Ocran, Kingsley Owusu Manu, Jo‐Ann Jackson, Pius Kyere, Lawrence Yourbebore Sobsah, Samuel Kyeremeh Adjei, Tampo Gyabong

**Affiliations:** ^1^ Department of Internal Medicine Methodist Hospital Wenchi Ghana; ^2^ Department of Radiology Holy Family Hospital Techiman Ghana

**Keywords:** case report, computed tomography, diaphragmatic eventration, elevated hemidiaphragm, syncope

## Abstract

Diaphragmatic eventration is a rare condition with an estimated incidence of less than 0.05% and is often discovered incidentally. Very rarely, symptomatic patients may present with syncope. Meticulous clinical assessment and appropriate imaging studies are crucial for unraveling the diagnosis.

## Graphical abstract

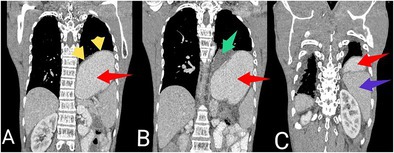

## 1 Introduction

The diaphragm is a large, dome‐shaped musculofibrous structure located at the base of the thoracic cavity. It is made up of the non‐contractile central tendon and muscle fibers that radiate centrifugally from the central tendon [1, 2]. The left and right hemidiaphragms are innervated by the ipsilateral phrenic nerves which originate from the anterior rami of the cervical nerve roots C3, C4, and C5 [3]. The diaphragm is the major inspiratory muscle of the body and also acts as a barrier, separating the thoracic and abdominal cavities [1, 2, 3].

Diaphragmatic eventration refers to a condition characterized by abnormal elevation of the entire or a portion of the hemidiaphragm due to nerve or muscle dysfunction while maintaining normal anatomical attachments [1]. It is a rare condition with an estimated incidence of less than 0.05% [1, 4]. The abnormality may be congenital or acquired. Congenital eventration is caused by a defect in the migration of myofibroblasts to the septum transversum during embryogenesis. This impairs diaphragmatic muscularization, resulting in partial or total replacement of the diaphragm muscle with fibroelastic tissue. It may also be associated with other congenital disorders such as spondylocostal dysostosis, Kabuki syndrome, Beckwith‐Wiedemann syndrome and Poland syndrome [1, 2]. Acquired cases are mostly seen in adults and can be caused by blunt or penetrating chest trauma, neck trauma, cervical spondylosis, cardiothoracic surgery, radiation therapy, connective tissue diseases and herpes zoster [2, 5].

Anatomically, both congenital and acquired diaphragmatic eventrations can be further classified as partial (involving only a part of the hemidiaphragm), complete (involving the entire hemidiaphragm), unilateral (involving one hemidiaphragm) or bilateral (involving both hemidiaphragms) [2]. Complete eventration usually occurs on the left side while partial eventration often involves the right hemidiaphragm [6]. Within the adult population, partial unilateral eventration is the most frequently encountered subtype which explains why the majority of affected individuals are asymptomatic [2]. These asymptomatic cases are discovered incidentally during evaluation for other conditions [7]. Patients who have complete or bilateral eventration, as well as those with comorbidities, are more likely to be symptomatic. Affected individuals may present with dyspnea, orthopnea, exertional dyspnea, chest pain, palpitations, chronic cough, recurrent respiratory infections, epigastric pain, dysphagia and heartburn [1, 8]. In instances where diaphragmatic eventration is complicated by gastric volvulus, patients may experience early satiety, vomiting, abdominal distension and constipation [9].

In this case report, we describe a rare manifestation of severe left diaphragmatic eventration in an elderly male who presented with recurrent syncope.

## 2 Case History and Examination

A 77‐year‐old male presented to the emergency unit of a primary care hospital in Ghana with a complaint of postprandial dyspnea which had persisted for 5 years. The dyspnea began insidiously and was aggravated by eating his fill or drinking too much water immediately after meals. It was associated with minimal exertional dyspnea, intermittent heartburn and orthopnea. There was however, no chest pain, palpitations, chronic cough, nausea, postprandial vomiting, epigastric pain or abdominal distension. Over the period, he was able to cope with his symptoms by reducing the size of his meals and also avoiding intake of too much water while eating. This resulted in unintentional weight loss of about 20 kg which was not associated with excessive sweating, tremors, diarrhea or heat intolerance. He sought medical care at several hospitals where he was frequently treated for suspected peptic ulcer disease with no resolution of his symptoms. On the day of presentation, he had sudden loss of consciousness which lasted about a minute. It was the second episode within a period of 3 months and was not preceded by an aura. Additionally, the episode was not associated with involuntary movements, foaming at the mouth, fecal incontinence, urinary incontinence or post‐event confusion. He had no previous history of hypertension, diabetes mellitus, ischemic heart disease, epilepsy, autoimmune diseases, recurrent pneumonia, herpes zoster or cervical spondylosis. He equally denied past history of neck or chest trauma. Again, he had never undergone any cardiothoracic or neck surgery in the past. He reported no prior exposure to radiation therapy for any malignancy. There was no family history of epilepsy, cardiovascular disease or other chronic illnesses. He occasionally drank alcohol but did not smoke cigarettes or use illicit drugs.

On physical examination, he was chronically ill‐looking, severely underweight with a body mass index of 16.9 kg/m^2^, afebrile (temperature = 37.1°C), anicteric, not pale, not in respiratory distress, not cyanosed and well hydrated. His oxygen saturation was 98% on room air. He was alert and oriented to person, place and time. There was no anterior neck swelling, tongue bite, oral thrush or peripheral lymphadenopathy. The pulse and blood pressure were 66 beats per minute and 134/78 mmHg, respectively. The respiratory rate was 18 cycles per minute with no chest wall deformity. In addition, there was no paradoxical chest movement. Chest expansion and tactile fremitus were reduced in the left lower lung zone. Percussion note was tympanic over the left lower lung zone with diminished air entry and audible gurgling sounds. The abdomen was scaphoid, soft and non‐tender with no organomegaly. Neurological assessment and precordial examination were unremarkable.

## 3 Differential Diagnosis, Investigations and Treatment

**FIGURE 1:**
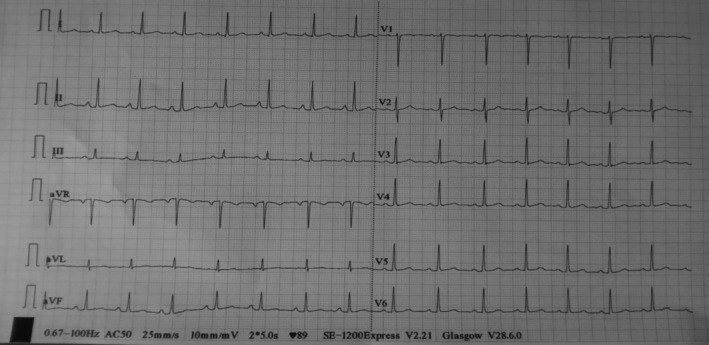
Resting 12‐lead ECG demonstrating normal sinus rhythm.

**FIGURE 2:**
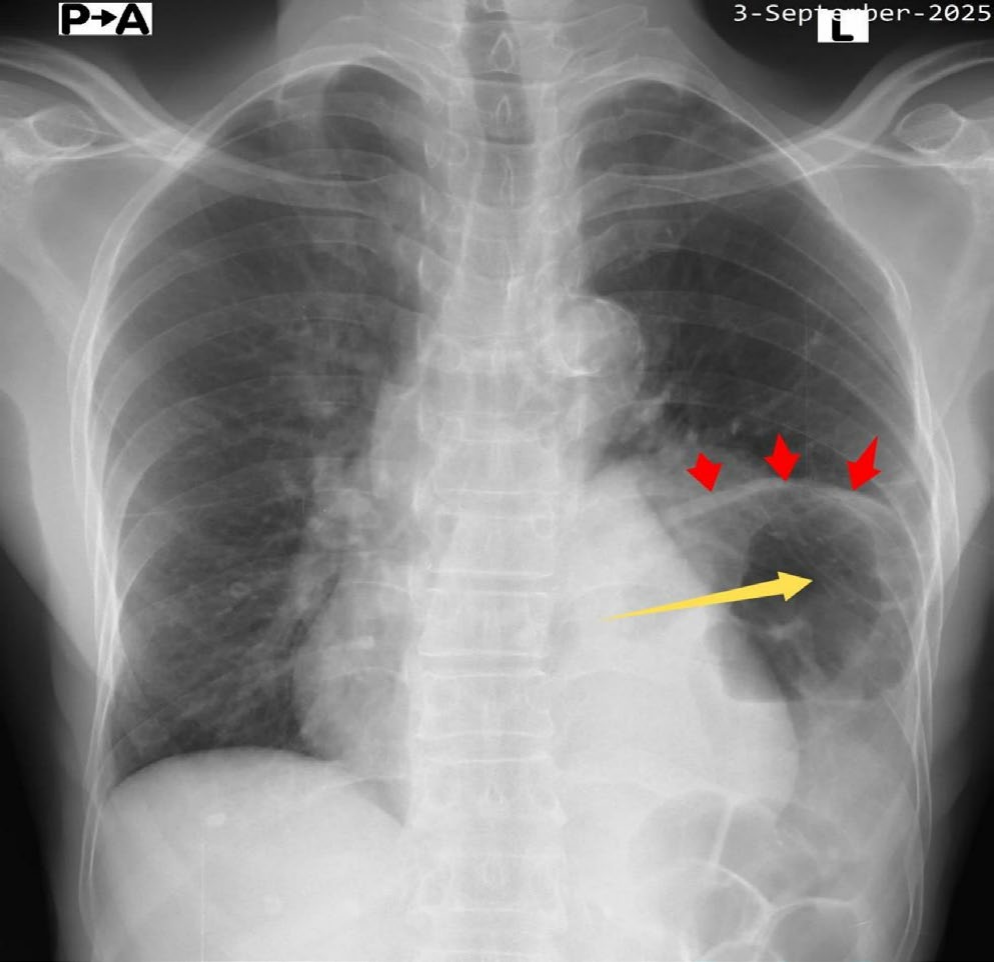
Erect PA chest X‐ray showing marked smooth elevation of the left hemidiaphragm (red arrows) with a large volume of air (yellow arrow) below the left hemidiaphragm.

**FIGURE 3:**
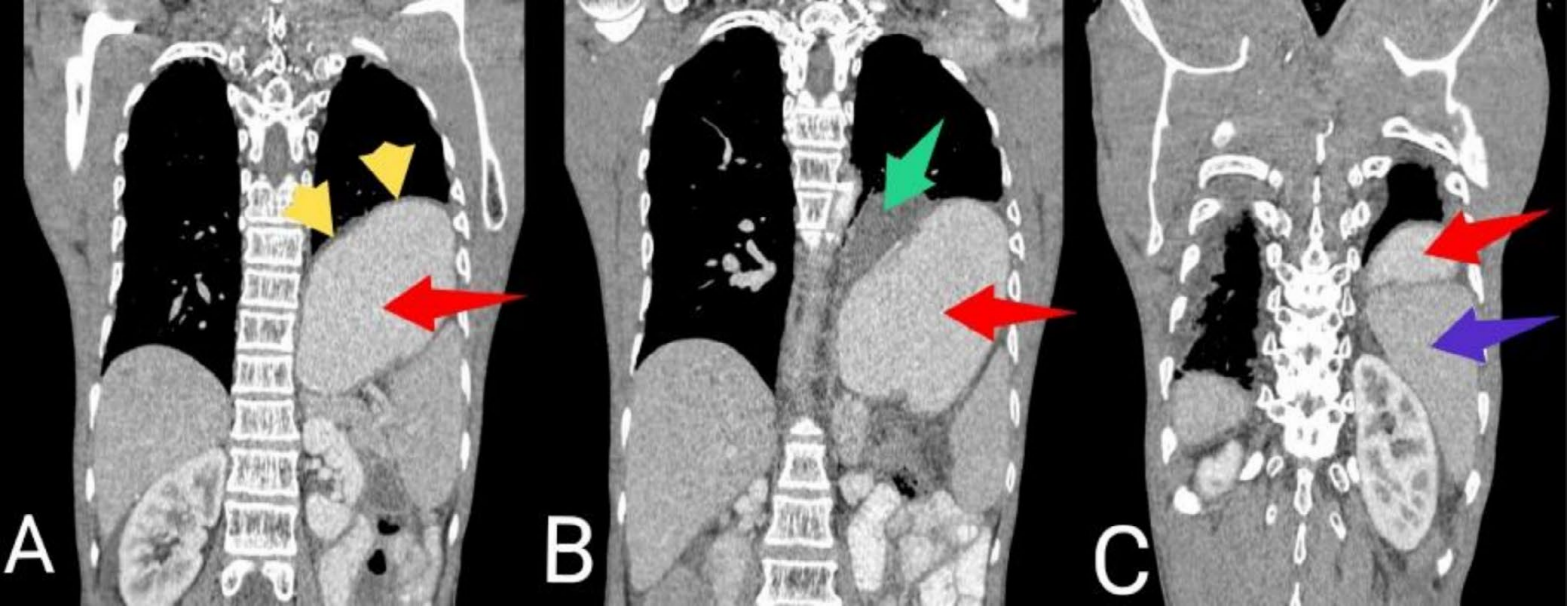
Contrast‐enhanced coronal chest CT scan images in mediastinal window showing significantly elevated intact smooth‐contoured left hemidiaphragm (yellow arrows in A) with upward displacement of the stomach (red arrows in A, B and C), mesenteric fat (green arrow in B) and spleen (blue arrow in C) into the left hemithorax.

**FIGURE 4:**
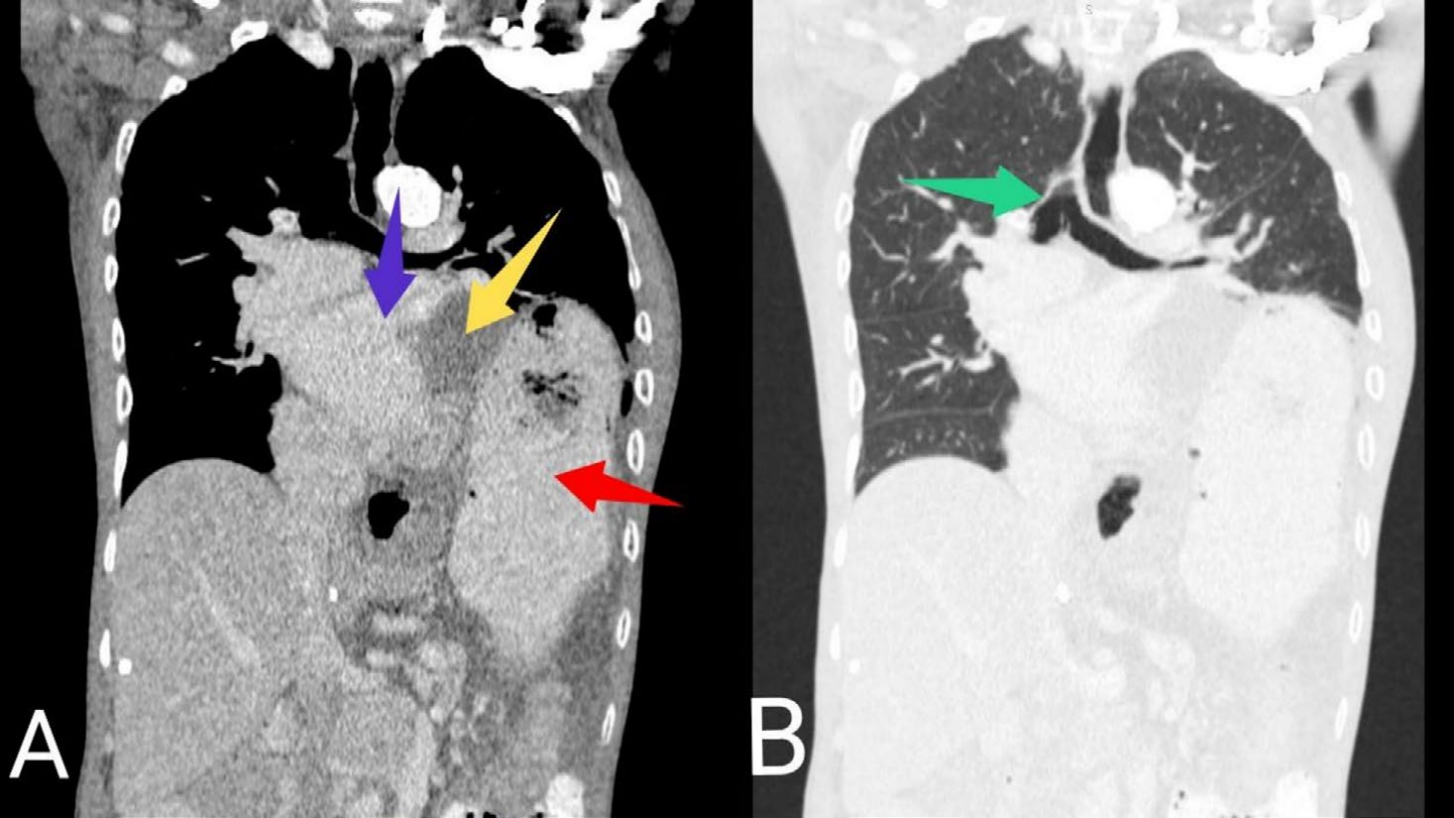
Contrast‐enhanced coronal chest CT scan images in mediastinal (A) and lung (B) windows showing the stomach (red arrow in A) and mesenteric fat (yellow arrow in A) directly compressing the heart (blue arrow in A) with associated minimal rightward mediastinal shift (green arrow in B).

His complete blood count, serum electrolytes, renal function test, liver biochemistries and thyroid function test were normal. Random blood glucose and glycated hemoglobin were 5.3 mmol/L and 4.5%, respectively. Serological test for human immunodeficiency virus was negative. Both resting 12‐lead electrocardiogram (ECG) (Figure 1) and Holter monitoring revealed normal sinus rhythm. Erect posteroanterior (PA) chest X‐ray demonstrated marked smooth elevation of the left hemidiaphragm with a large volume of air beneath it (Figure 2). At this point, our differential diagnoses were left diaphragmatic eventration, left diaphragmatic hernia, left diaphragmatic paralysis and left‐sided Chilaiditi's syndrome, necessitating further evaluation with contrast‐enhanced computed tomography (CT) scan of the chest. The CT scan showed significant elevation of the intact smooth‐contoured left hemidiaphragm with upward displacement of the stomach, mesenteric fat and spleen into the left hemithorax (Figure 3). Also noted were the stomach and mesenteric fat directly compressing the heart with attendant minimal rightward mediastinal shift (Figure 4). In addition, there was atelectasis of the lower lobe of the left lung. Subsequent echocardiographic examination revealed left and right ventricular diastolic dysfunction with left ventricular ejection fraction of 59.89%. Fluoroscopic sniff testing and pulmonary function test were not performed because they were not readily available in our hospital or any of the other facilities in our region.

A diagnosis of severe left diaphragmatic eventration was made. The patient was educated about the condition. He was advised to eat smaller, more frequent meals (i.e., five or six smaller meals per day) and also increase intake of high‐calorie and protein‐rich foods. Additionally, he was encouraged to sit upright while eating and avoid lying down for at least 30 min after eating. Furthermore, he was counseled to stay well hydrated, but to consider reducing water intake during meals if it made him feel excessively full. Equally, he was told to avoid carbonated drinks, gas‐producing foods and to minimize engagement in strenuous physical activities. The need for surgical intervention was extensively discussed with the patient and his relatives.

## 4 Outcome and Follow‐Up

There was no recurrence of syncope throughout the period of admission. Also, he reported significant improvement in postprandial dyspnea with adherence to dietary counseling and supportive care. Following discharge after 10 days of hospitalization, he was referred to a tertiary hospital to see a cardiothoracic surgeon for further assessment and possible surgical plication.

## 5 Discussion

Painstaking clinical assessment of symptomatic patients and appropriate imaging studies are crucial for the prompt and accurate diagnosis of diaphragmatic eventration. The rarity of this diaphragm abnormality and its non‐specific symptoms may lead to delayed or misdiagnosis which can have serious consequences for affected individuals as was noted in the patient described in this case report.

The age of the patient and the duration of his symptoms strongly suggest that the eventration was acquired. Acquired diaphragmatic eventration commonly occurs in adults and is often due to phrenic nerve injury with resultant muscle atrophy [2]. As was observed in this patient, the cause of an old phrenic nerve injury leading to the development of diaphragmatic eventration may not be immediately apparent or may be remote [2]. The phrenic nerve injury could have occurred many years prior to the onset of symptoms, making it extremely difficult to trace the root cause. Approximately 20% of phrenic nerve injuries resulting in diaphragm dysfunction remain idiopathic despite extensive investigations [10].

The patient had a long‐standing history of postprandial dyspnea which was associated with orthopnea, exertional dyspnea and heartburn. As demonstrated by chest imaging, he had severe left diaphragmatic eventration with upward displacement of the stomach, spleen and mesenteric fat into the ipsilateral hemithorax. Postprandially, the distended stomach pushes the high‐riding left hemidiaphragm further into the thoracic cavity, restricting lung expansion and causing shortness of breath.

Additionally, our patient experienced recurrent syncopal episodes. However, there is limited literature on a direct causal link between diaphragmatic eventration and syncope. The association between these two conditions has primarily been described in only a few case reports [5, 11], often in the context of severe eventrations, such as the one identified in this patient. A number of plausible pathophysiologic mechanisms may be responsible for this unusual presentation. Firstly, the mediastinal shift associated with severe diaphragmatic eventration can trigger cardiac arrhythmias [1], which may lead to inadequate cardiac output and, subsequently, syncope. Secondly, it has been suggested that cardiac compression occurring in severe diaphragmatic eventration can impair the ability of the cardiac chambers to relax and fill with blood during diastole [5, 11, 12]. This culminates in reduced cardiac output, which may eventually lead to cerebral hypoperfusion and syncope. Also, the vagal reflex triggered by the act of swallowing or a distended stomach in the chest can cause severe bradycardia, hypotension and peripheral vasodilation, resulting in a transient loss of consciousness [13]. As noted, resting 12‐lead ECG and Holter monitoring showed normal sinus rhythm, which essentially ruled out cardiac arrhythmia as the possible cause of syncope in this patient. This prompted further evaluation with echocardiography. The ventricular diastolic dysfunction detected during echocardiographic examination was likely due to the mechanical effects of the cranially displaced abdominal organs and the associated mediastinal shift. This may have led to low cardiac output with resultant cerebral hypoperfusion, causing recurrent syncope in our patient.

Similar to our case report, Xia et al. described a 45‐year‐old man who presented with a 1‐year history of recurrent syncope. Chest CT scan confirmed severe left diaphragmatic eventration with cephalic displacement of the stomach, spleen, colon and pancreas into the left hemithorax. His syncopal events were attributed to the compression of cardiac chambers, leading to a reduction in cardiac output [11]. In another report, a 65‐year‐old female complained of recurrent syncope. After extensive investigations, she was diagnosed with eventration of the left hemidiaphragm. It was thought that a transient decrease in cardiac output, caused by left ventricle compression and mediastinal shift, was partly responsible for the syncope [5]. Generally, the scarcity of literature and data relating to the precise relationship between diaphragmatic eventration and syncope calls for further research in this area.

During the clinical assessment of the patient, a couple of differential diagnoses were considered. While an erect PA chest X‐ray can demonstrate elevation of the hemidiaphragm as seen in this case, advanced skills are necessary to interpret findings from more specialized studies in order to differentiate diaphragmatic eventration from other conditions with similar chest X‐ray findings. In this patient, the chest CT scan did not show any defect in the diaphragm, thereby ruling out diaphragmatic hernia. Fluoroscopic sniff testing can differentiate diaphragmatic eventration from paralysis, as the latter may cause paradoxical motion during sniffing [1]. This test was not performed for the patient because it was unavailable. Some clinicians have argued that the distinction between diaphragmatic eventration and paralysis is irrelevant because both conditions are treated with surgical plication when conservative management fails [1]. Last but not least, left‐sided Chilaiditi's syndrome was also contemplated. Chilaiditi's syndrome is typically characterized by the interposition of the colon between the liver and the right hemidiaphragm with associated clinical symptoms [14]. Occasionally, it can occur on the left side [15]. Chest CT scan however, did not show any loop of colon trapped between the left hemidiaphragm and the stomach or spleen.

The management of diaphragmatic eventration depends on the severity of the condition. Conservative management or supportive care is recommended for asymptomatic individuals as well as those with mild symptoms [1, 2]. As evident from the physical assessment, the patient was severely underweight and was therefore offered adequate nutritional counseling. This is critically important, especially for patients who are underweight and preparing for surgery. Adequate preoperative nutritional support is necessary for improving surgical outcomes and enhancing postoperative patient recovery [16]. Eating smaller, more frequent meals also prevents the stomach from becoming overly distended and causing postprandial dyspnea. Furthermore, sitting upright for an extended period after meals and avoiding gas‐producing foods as well as carbonated drinks are crucial supportive measures. These strategies help alleviate symptoms and improve the overall quality of life of the patient, particularly in instances where surgical intervention is delayed or not immediately feasible, as was the case in our patient.

Surgical plication is the definitive treatment for patients with severe diaphragmatic eventration. This surgical procedure flattens and lowers the abnormally elevated hemidiaphragm which allows the affected lung to re‐expand and increase total lung capacity, thus making breathing easier and more efficient. It can be performed via open thoracotomy and minimally invasive approaches such as video‐assisted thoracoscopic surgery (VATS), laparoscopic, or robotic‐assisted surgery [1, 17]. Our patient had severe symptoms and was extensively counseled on the need for surgical intervention. Due to human resource constraints in our hospital, he was referred to see a cardiothoracic surgeon at a tertiary center for possible diaphragmatic plication.

## 6 Limitation

Information regarding the patient's treatment and follow‐up at the tertiary hospital was not included in this case report because it was not available to us.

## 7 Conclusion

Rarely, diaphragmatic eventration may present with syncope. While many asymptomatic cases are discovered incidentally, meticulous clinical evaluation and appropriate imaging studies are crucial for unraveling the diagnosis in symptomatic patients.

## Author Contributions

**Prosper Adjei:** conceptualization, data curation, investigation, writing – original draft, writing – review and editing. **Edward Ebo Ocran:** investigation. **Kingsley Owusu Manu:** data curation. **Jo‐Ann Jackson:** data curation. **Pius Kyere:** data curation. **Lawrence Yourbebore Sobsah:** data curation. **Samuel Kyeremeh Adjei:** writing – review and editing. **Tampo Gyabong:** data curation.

## Ethics Statement

The authors have nothing to report.

## Consent

Written informed consent was obtained from the patient to publish this report in accordance with the journal's patient consent policy.

## Conflicts of Interest

The authors declare no conflicts of interest.

## Data Availability

Data sharing is not applicable.
